# The complete chloroplast genome of *Pseudosasa usawae* (Poaceae: Bambusoideae: Arundinarieae) in Taiwan

**DOI:** 10.1080/23802359.2022.2026829

**Published:** 2022-01-27

**Authors:** Kun-Cheng Chang, Kuan-Ning Kung, Yu-Chun Hsu, Tsung-Po Chang, Shui-Lin Deng

**Affiliations:** a Department of Forestry and Nature Resources, National Chiayi University, Chiayi, Taiwan; b Chungpu Research Center, Taiwan Forestry Research Institue, Chiyai, Taiwan; c Department of Agriculture Science, National Chiayi University, Chiayi, Taiwan

**Keywords:** *Arundinarieae*, *Bambuosideae*, complete chloroplast genome, *Pseudosasa usawae*

## Abstract

*Pseudosasa usawae* is an endemic species in Taiwan, and grows at an altitude of 600–1200 m. In this study, we fully characterized the complete chloroplast genome of *P. usawae*. The complete chloroplast sequence was 139,660 bp, including large single-copy (LSC), small single-copy (SSC), and a pair of invert repeats (IR) region of 83,271, 12,803, and 21,793 bp. Besides, the plastid genome comprised a total of 129 genes, including protein-coding, tRNA, and rRNA genes as 83, 38, and 8 genes. Phylogenetic analysis reveals that *P. usawae* is closely associated with Phyllostachys genus clade, sister to the lineage of *Phyllostachys*.

*Pseudosasa usawae* (Hayata) Makino and Nemoto 1931, a temperate woody bamboo, is a valuable edible bamboo for its shoots in the family Poaceae (Bambusoideae). This species grows at altitudes from 600 to 1200 m which is scattered and associated with broadleaved forests thought the island (Lin 1978; Lin 2000). In the past, *P. usawae* was often classified as a species in genus of *Arundinaria* (Hayata 1916), *Pleioblastus* (Ohki 1928), or *Pseudosasa* (Makino and Nemoto 1931). For a long time, Botanist focused on morphological and anatomical to identify the species of Bambusoideae (Leandro et al. 2020). In the recent years, molecular biological methods have been used to give detailed information about the phylogenetic of the Bambusoideae, such as *Ampelocalamus naibuensis* (Zhang and Chen 2016), *Gelidocalamus xunwuensis* (Zhang et al. 2019), *Acidosasa gigantea* (Zheng et al. 2020), *Bambusa stenoaurita* (Xia et al. 2021), but not *P. usawae*. To date, the genetic data of bamboos of Taiwan are very scarce. In this study, we obtained and first reported the complete chloroplast genome of *P. usawae*.

Fresh leaves as sequencing materials were collected from Taipei Botanical Garden, Taiwan forestry research institute in Taiwan (25°01′54.7″N 121°30′33.3″E). The collection followed the guidelines of the specimen collection of Taiwan Forestry Research Institute. The voucher specimen was deposited at the Herbarium of Department of Forestry and Natural Resources, National Chiayi University, Taiwan (Herbarium code: CHIA, Kun-Cheng Chang/kcchang@mail.ncyu.edu.tw) under collection number of K. N. Kung 3149. High-throughput sequencing library preparation and sequencing using the Illumina NovaSeq system (2 × 150 bp paired-end) were performed by Tri-I Biotech, Inc. in New Taipei city, Taiwan. The complete chloroplast genome was performed using CLC Genomic workbench 21 (CLC Inc., Aarhus, Demark) with *Pseudosasa cantorii* (NC036824) chloroplast genome as the reference sequence. The annotation was predicted by using GeSeq in default settings (Tillich et al. 2017) and was deposited in GenBank under the accession number of MZ153211.

The complete chloroplast genome of sample *P. usawae* is 139,660 bp, a mean coverage of 321.4. The chloroplast genome contained a pair of inverted repeats (21,793 bp each) separated by a large single-copy region (LSC) of 83,271 bp and a small single-copy region (SSC) of 12,803 bp. The overall base composition is 30.6% for A, 30.5% for T, 19.3% for C, 19.6% for G. The GC content of the whole genome is 38.9%. The genome encoded 129 genes, consisting of 83 coding genes, 38 tRNA genes and 8 rRNA genes.

To evaluate the phylogenetic position of *P. usawae*, additional 37 complete chloroplast genomes in the tribe (Figure 1). *Bambusa emeiensis* NC015830, *Dendrocalamus latiflorus* NC013088 and *Chusquea circinata* NC024790, the three species in Bambuseae as outgroups, were retrieved from NCBI. All sequences were aligned with MAFFT (Katoh and Standley 2013). The maximum-likelihood tree was constructed using 1000 bootstrap replicates by RAxML (Stamatakis 2014). The results showed that *P. usawae* is distantly related to *P. cantorii*, which is clustered in clade (VI). *P. usawae* is clustered in the clade (V) with high-support value, as a sister group with the lineage of *Phyllostachys* genus. The internodes are short between these lineages in the phylogenetic tree, reflecting the probable recent rapid radiation in clade (V).

**Figure 1.**
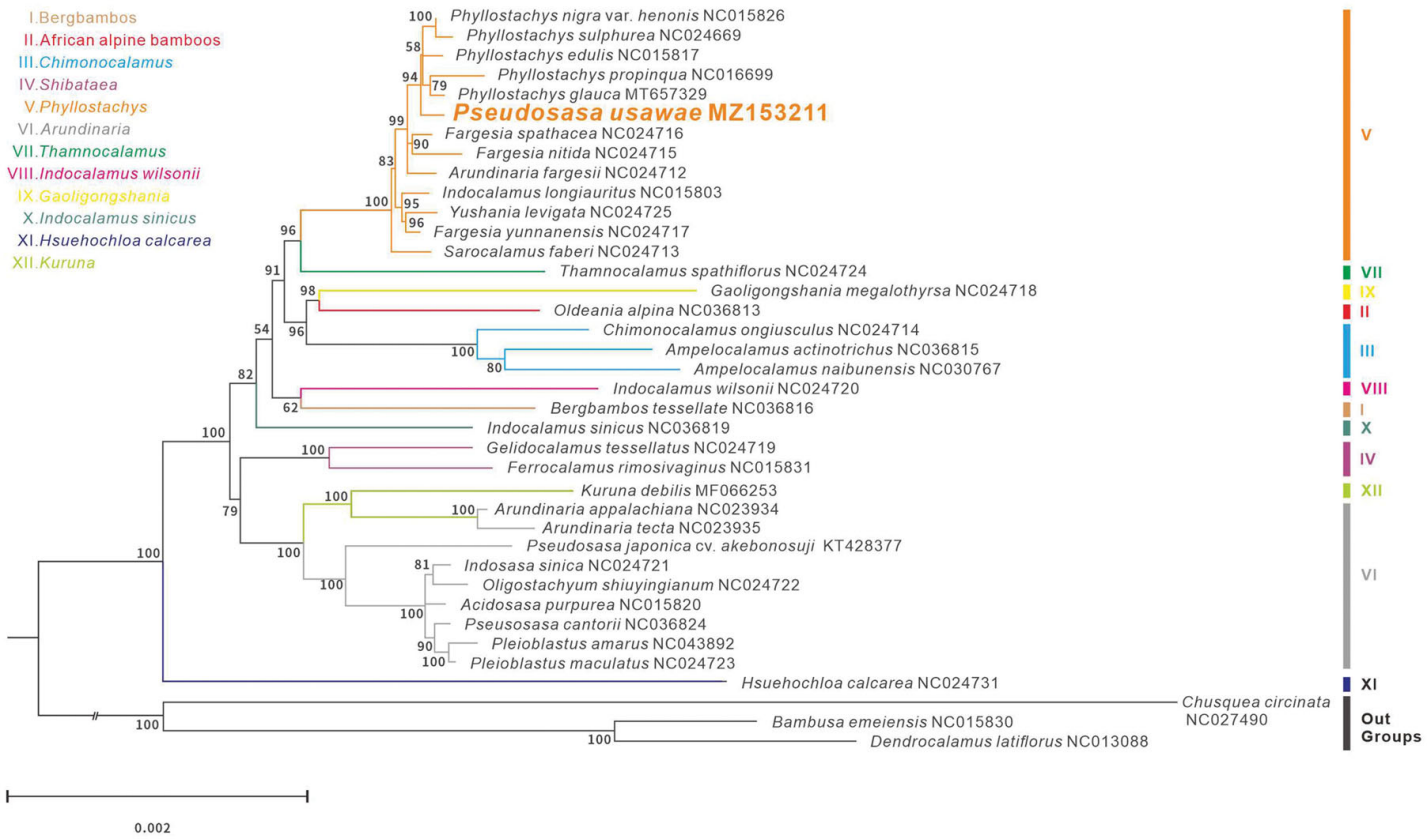
Phylogeny of *Pseudosasa usawae* and 37 species of Bambusoideae were constructed using the maximum likelihood (ML) method by analyzing the chloroplast complete genome. Numbers above each branch are the ML bootstrap support.

## Data Availability

The genome sequence data that support the findings of this study are openly available in GenBank of NCBI at https://www.ncbi.nlm.nih.gov/ under the accession no.MZ153211. The associated BioProject, SRA, and Bio-Sample numbers are PRJNA738262, SRR14826263, and SAMN19716639 respectively.
